# Bibliometric analysis of pyroptosis in pathogenesis and treatment of acute lung injury

**DOI:** 10.3389/fmed.2024.1488796

**Published:** 2025-01-22

**Authors:** Chun Wang, Na Liu

**Affiliations:** Department of Anesthesiology, The Second Affiliated Hospital of Dalian Medical University, Dalian, Liaoning, China

**Keywords:** acute lung injury, pyroptosis, inflammasome, Nrf2 signaling pathway, targeted therapy

## Abstract

**Objective:**

This study aims to conduct a bibliometric analysis to assess the present state, thematic focus, and emerging developments in the research literature on the involvement of pyroptosis in the pathogenesis and treatment of acute lung injury (PFALI), as well as other pertinent research areas.

**Methods:**

This bibliometric study examined PFALI research published from 1 January 2004 to 24 May 2024, utilizing the Web of Science database. The analysis was conducted using CiteSpace, VOSviewer, R, and GraphPad Prism 8.0, and encompassed metadata on the countries, institutions, authors, journals, and keywords represented in the literature.

**Results:**

This study analyzed 1,495 publications, comprising 1,194 articles and 301 reviews, to assess the publication output on PFALI. China exhibited the highest output with 964 (64.48%) articles. Central South University was the most prolific institution, contributing 54 (3.61%) publications. Zhou, Yong had the greatest individual publication record, with 15 (3.59%) articles. The journal International Immunopharmacology published the most PFALI-related articles at 76 (5.09%). The identified research frontiers for upcoming years include “iron,” “sirt1,” “repair,” and “alveolar macrophage pyroptosis.”

**Conclusion:**

This bibliometric analysis comprehensively examined research trends and advancements related to PFALI, including the contributions of key authors, institutions, and countries.

## Introduction

Acute lung injury (ALI) is an acute, progressive inflammatory condition of the lungs characterized by inflammatory cell infiltration, increased permeability of the alveolar-capillary barrier, and acute diffuse alveolar edema. It is a life-threatening hypoxic respiratory disease (1). Pyroptosis is a newly discovered form of programmed cell death that depends on the cleavage of the gasdermin (GSDM) protein by caspases in the cytoplasm. The activated N-terminal domain of GSDM (GSDM-NTD) forms pores in the cell membrane, ultimately leading to cell swelling, lysis, and amplified inflammatory responses (2–4). While apoptosis is a physiological, genetically regulated form of cell death characterized by membrane blebbing, cell shrinkage, and formation of apoptotic bodies without cell rupture or inflammatory factor release (5, 6). Growing evidence suggests that pyroptosis plays a crucial role in the pulmonary inflammatory response in ALI (7–9). Inhibiting pyroptosis is a promising therapeutic approach for ALI.

Bibliometric analysis is a well-established approach to evaluate published research and identify emerging trends in scientific fields. This quantitative method utilizes mathematical and statistical techniques to investigate various aspects of academic disciplines, including collaborations among countries, institutions, authors, journals, and individual publications (10–12). In the context of ALI, there has been a substantial volume of research focused on the role of pyroptosis in the pathogenesis and treatment of this condition. However, a comprehensive bibliometric assessment of the literature on pyroptosis in the pathogenesis and treatment of acute lung injury (PFALI) is currently lacking. Such an analysis could provide valuable insights into the evolving landscape of this research domain. The present article will use bibliometric methods to provide an overview of the latest advances in the involvement of the pyroptosis mechanism in the inflammatory response of ALI, with the aim of offering new insights into the pathogenesis and treatment of ALI.

## Materials and methods

### Search strategy

On 24 May 2024, a comprehensive literature search was conducted in the Web of Science Core Collection (WoSCC) at The Second Affiliated Hospital of Dalian Medical University. The search strategy employed the following query: ((((TS = (Inflammasome OR Pyroptosis)) AND TS = (Acute Lung Injury OR Lung Injury OR ARDS OR Acute Respiratory Distress Syndrome)) AND LA = (English)) AND DT = (Article OR Review)) NOT DT = (Retracted Publication OR Proceedings Paper OR Book Chapters). This search retrieved articles that referenced PFALI or its synonyms in the title, abstract, or keywords. The search was limited to English-language articles and review papers published between 1 January 2004 and 24 May 2024, excluding documents predating 1 January 2004, as well as case reports, meeting abstracts, editorial materials, and other non-article/review document types.

### Data collection

On 24 May 2024, a literature search was conducted in the WoSCC to retrieve comprehensive data on PFALI-related research. The bibliometric data collected included authorship details, publication titles, source information, funding acknowledgments, citation counts, abstracts, author affiliations, document types, and cited references. The data was obtained in various formats, such as txt, Excel, tab, and BibTeX, to enable further analysis. Additionally, the H-index of the top 10 most prolific authors and the 2022 impact factor and Journal Citation Report category quartile of the 10 primary PFALI-focused journals were sourced from Web of Science. This extensive data collection aimed to provide a robust foundation for the subsequent bibliometric analysis and visualization of the PFALI research landscape.

### Statistical analysis

The bibliometric data was analyzed using an array of specialized software tools, including VOSviewer 1.6.19, CiteSpace 6.1R6, SCImago Graphica Beta 1.0.3, and the bibliometrix package (version 4.1.2) in R language (version 4.2.3). VOSviewer was employed to identify the top 10 keywords with the highest occurrence and to cluster the leading 46 keywords. Utilizing VOSviewer, Pajek64, and SCImago Graphica, knowledge maps were created to visualize prominent authors, contributing nations and institutions, core journals, influential publications, co-occurring keywords, and co-cited references. CiteSpace was used to evaluate the collaborative centrality among countries/regions, institutions, and authors. Furthermore, the bibliometrix package was leveraged for trend topic detection and to produce visual depictions of publication volume and collaborative relationship networks. This comprehensive suite of bibliometric analyses and visualization techniques provided a robust and multifaceted examination of the PFALI research landscape.

## Results

### Overview

**FIGURE 1:**
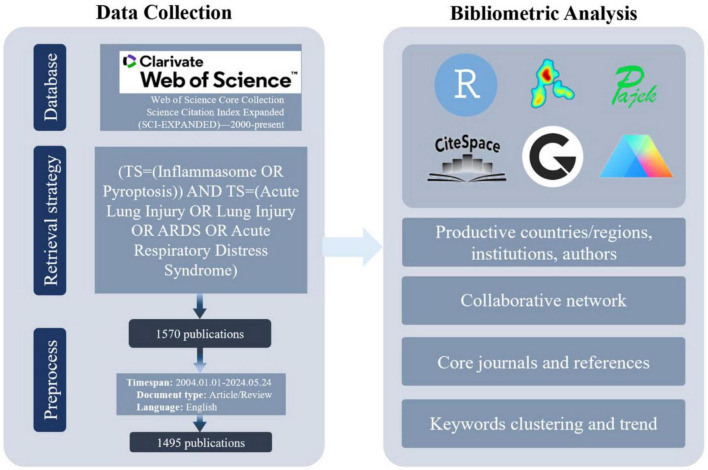
Hierarchical chart depicting the process of publication selection.

The comprehensive literature search of the WoSCC database yielded a total of 1,495 publications related to PFALI, covering the period from 1 January 2004 to 24 May 2024. These publications comprised 1,194 original research articles and 301 review articles, as depicted in Figure 1.

**FIGURE 2:**
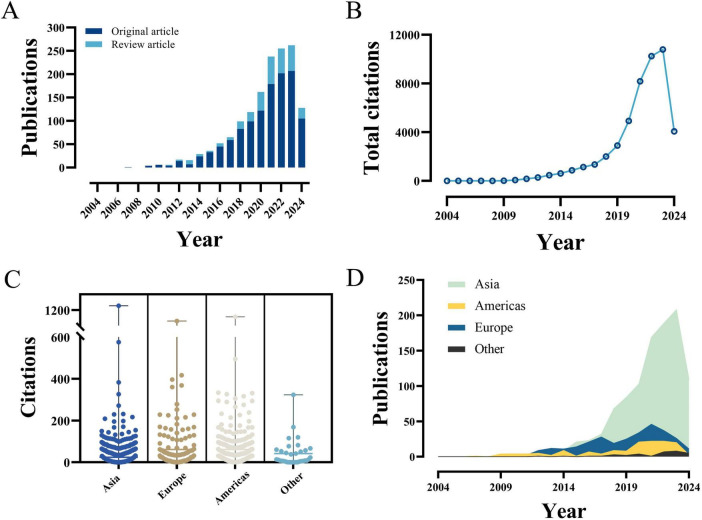
The yearly quantity and citations of publications pertaining to PFALI. **(A)** The yearly quantity. **(B)** The yearly citations. **(C)** The yearly citations to each continent. **(D)** The yearly quantity to each continent.

The frequency of PFALI-related publications exhibited an increasing pattern over time, meanwhile, accompanied by an overall upward trend in total citations, as shown in Figures 2A, B. Furthermore, the compiled data on total citations and annual publications by continent are presented in Figures 2C, D. Collectively, the PFALI-related publications have amassed a total of 48,158 citations, resulting in an average of 32.78 citations per article. This remarkable citation count underscores the significant impact and importance of this research field within the scientific community.

### Leading countries/regions

**FIGURE 3:**
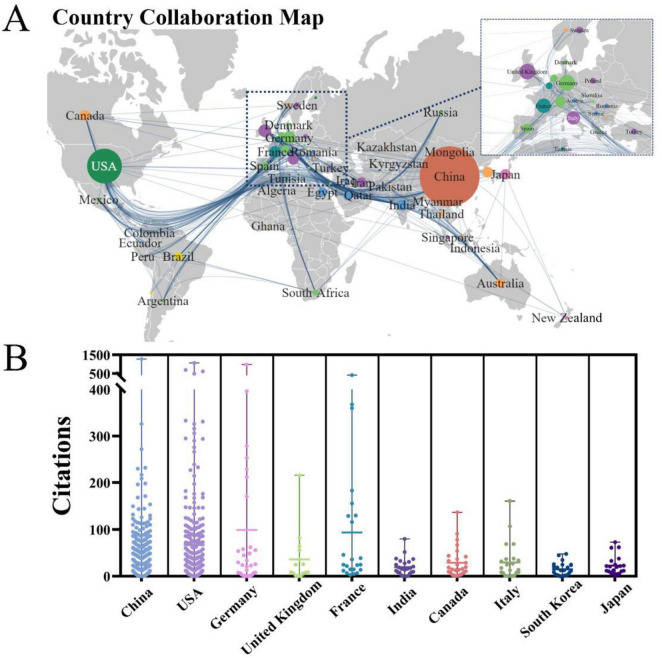
The collaborative map and clusters of the country. **(A)** Country collaboration map and documents density. **(B)** Visualization of publication output among major countries.

The PFALI-related publications were disseminated across 62 countries/regions spanning six continents, with notable collaboration observed among the regions of East Asia, North America, and Western Europe, as depicted in Figure 3A. The top five countries ranked by productivity in publishing PFALI articles are: China with 964 publications (64.48%), the United States with 324 publications (21.67%), Germany with 44 publications (2.94%), the United Kingdom with 43 publications (2.88%), and France with 36 publications (2.41%). Regarding citation impact, the articles originating from China have received the highest total number of citations at 21,785, while those from Germany have the highest average number of citations per article at 76.48, as shown in Table 1. The citation patterns across the contributing countries were further visualized in Figure 3B.

**TABLE 1:** The top 10 countries/regions with the highest productivity.

Rank	Country	Publications *n* (%)	Total citations	Average citations	Collaborative centrality
1	China	964 (64.48)	21,785	22.60	0.33
2	United States	324 (21.67)	18,658	57.59	0.45
3	Germany	44 (2.94)	3,365	76.48	0.10
4	United Kingdom	43 (2.88)	1,399	32.53	0.14
5	France	36 (2.41)	2,473	68.69	0.09
6	India	33 (2.21)	528	16.00	0.19
7	Canada	31 (2.07)	1,028	33.16	0.06
8	Italy	31 (2.07)	809	26.10	0.01
9	South Korea	30 (2.01)	599	19.97	0.00
10	Japan	29 (1.94)	905	31.21	0.06

The observed patterns of collaboration and citation influence shed light on the global collaborative networks and intellectual structure underpinning the PFALI research domain.

### Active institutions and authors

The comprehensive analysis of the PFALI research landscape identified a total of 1,495 publications authored by 8,806 individuals affiliated with 1,629 institutes across 62 countries and regions. The top five most prolific institutions contributing to this body of research are: Central South University (54 publications, 3.61%), Fudan University (50 publications, 3.34%), Shanghai Jiao Tong University (47 publications, 3.14%), Huazhong University of Science and Technology (38 publications, 2.54%), and Nanjing Medical University (36 publications, 2.41%), as shown in Table 2.

**TABLE 2:** The top 10 productive institutions.

Rank	Institution	Country	Publications *n* (%)	Total citation	Average citation
1	Central South University	China	54 (3.61)	1,747	32.35
2	Fudan University	China	50 (3.34)	1,500	30.00
3	Shanghai Jiao Tong University	China	47 (3.14)	1,553	33.04
4	Huazhong University of Science and Technology	China	38 (2.54)	839	22.08
5	Nanjing Medical University	China	36 (2.41)	761	21.14
6	Wuhan University	China	36 (2.41)	1,105	30.69
7	Zhejiang University	China	31 (2.07)	1,726	55.68
8	Shandong University	China	28 (1.87)	625	22.32
9	Tongji University	China	26 (1.74)	595	22.88
10	Wenzhou Medical University	China	23 (1.54)	386	16.78

**FIGURE 4:**
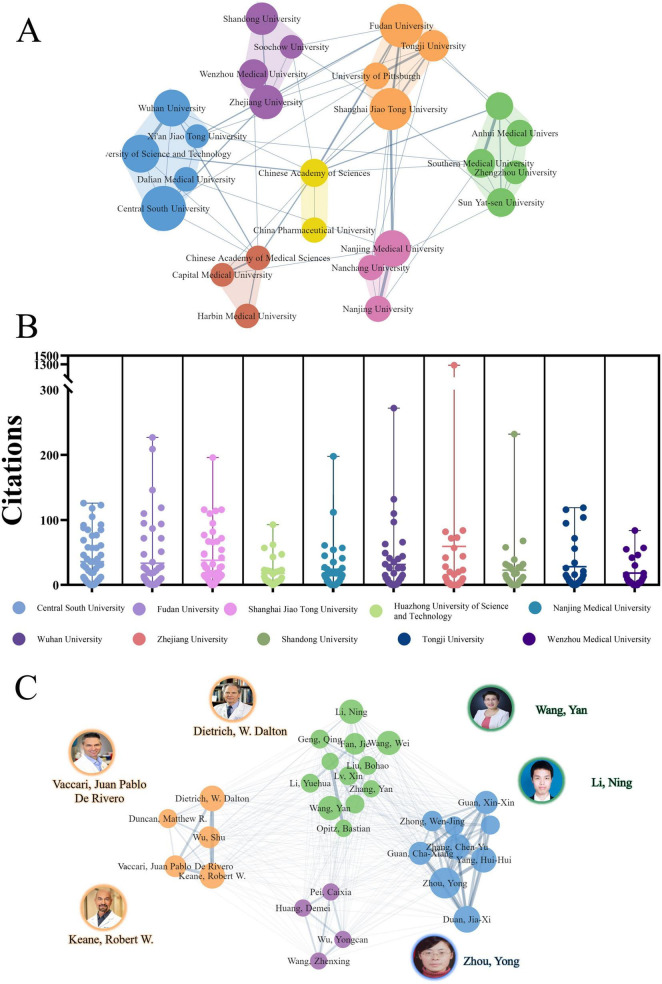
Collaborative clustering of institutions and authors. **(A)** Collaborative clustering of institutions. **(B)** Citations to each institute. **(C)** Collaborative clustering of authors.

The institutional collaboration network revealed seven distinct clusters, with the cluster consisting of Central South University, Fudan University, Nanjing Medical University, and others demonstrating the highest degree of collaboration, as depicted in Figure 4A. The citation patterns for the top institutions were further visualized in Figure 4B, providing insights into the intellectual influence and impact of these leading research centers within the PFALI domain. Among the top 10 most productive authors, Zhou, Yong was the most prolific with 15 publications (3.59%), followed by Carvajal, Richard D. with 11 publications and Johnson, Douglas B. with 16 publications (1.07%). Notably, 6 of the top 10 most productive authors were affiliated with institutions in China, while the remaining 4 were from the United States. Dietrich, W. Dalton from the University of Miami in the United States exhibited the highest h-index of 93 among the top authors, underscoring his significant scholarly impact (Table 3).

**TABLE 3:** The top 10 productive authors.

Rank	Author	Institution	Country	Publications *n* (%)	Total citation	Average citation	H-index
1	Zhou, Yong	Central South University	China	16 (1.07)	682	42.63	20
2	Duan, Jia-Xi	Central South University	China	12 (0.80)	514	42.83	18
3	Dietrich, W. Dalton	University of Miami	United States	11 (0.74)	339	30.82	93
4	Keane, Robert W.	University of Miami	United States	11 (0.74)	326	29.64	45
5	Yang, Hui-Hui	Shenzhen Technology University	China	11 (0.74)	466	42.36	21
6	Li, Ning	Wuhan University	China	10 (0.67)	156	15.60	9
7	Wang, Yan	Shanghai Jiao Tong University	China	10 (0.67)	237	23.70	14
8	Guan, Cha-Xiang	Central South University	China	9 (0.60)	469	52.11	22
9	Fan, Jie	University of Pittsburgh	United States	8 (0.54)	661	82.63	38
10	Vaccari, Juan Pablo De Rivero	University of Miami	United States	8 (0.54)	221	27.63	33

The authors exhibited a relatively elevated level of cooperation, as evidenced by the presence of seven clusters in the collaborative network visualized relatively elevated level of cooperation, as evidenced by the presence of seven clusters, as depicted in Figure 4C. This comprehensive analysis of the PFALI research landscape, encompassing the institutional, authorial, and collaborative dimensions, provides a rich and multifaceted understanding of the intellectual structure, productivity, and impact within this important field of study.

### Core journals and references

The expansive PFALI research has been disseminated across 445 academic journals, with the top 10 most productive journals presented in Table 4.

**TABLE 4:** The top 10 core journals.

Rank	Journal	Publications *n* (%)	Total citations	Average citations	2022 JCR category quartile	2022 IF
1	International Immunopharmacology	76 (5.09)	1,441	18.96	Q2	5.6
2	Frontiers in Immunology	62 (4.15)	2,611	42.11	Q1	7.3
3	International Journal of Molecular Sciences	36 (2.41)	1,497	41.58	Q1	5.6
4	Biomedicine & Pharmacotherapy	28 (1.87)	674	24.07	Q1	7.5
5	Inflammation	28 (1.87)	821	29.32	Q2	5.1
6	Journal of Immunology	27 (1.81)	2,188	81.04	Q2	4.4
7	Frontiers in Pharmacology	26 (1.74)	747	28.73	Q1	5.6
8	American Journal of Physiology – Lung Cellular and Molecular Physiology	23 (1.54)	739	32.13	Q1	4.9
9	Journal of Ethnopharmacology	21 (1.40)	161	7.67	Q1	5.4
10	Life Sciences	21 (1.40)	556	26.48	Q1	6.1

The journal exhibiting the highest level of productivity is International Immunopharmacology, which has published 76 (5.09%) PFALI-related articles, followed by Frontiers in Immunology with 62 (4.15%) articles, International Journal of Molecular Sciences with 36 (2.41%) articles, Biomedicine & Pharmacotherapy with 28 (1.87%) articles, and Inflammation with an additional 28 (1.87%) articles (Table 4). Applying the bibliometric principle of Bradford’s Law, which describes the distribution of scientific literature, a core set of 13 journals has been identified as the most influential in the PFALI research domain, including the aforementioned top publishing outlets as well as others (Supplementary Figure 1A). The citation patterns for these core journals were further visualized (Supplementary Figure 1B), providing insights into their relative intellectual impact within the field. Notably, the Journal of Immunology has achieved the highest average citation rate among the PFALI publications, with an average of 81.04 citations per article. The top 10 most-cited PFALI publications, as listed in Table 5, underscores the significant influence and impact of these seminal works within the research community.

**TABLE 5:** The top 10 core literatures with the highest citations.

Rank	First author	Title	Journal	Type	Year of publication	Total citations
1	Ahmed, Syed Minhaj Uddin (13)	Nrf2 signaling pathway: pivotal roles in inflammation	Biochimica et Biophysica Acta – Molecular Basis of Disease	Review	2017	1,282
2	Wynn, Thomas A. (14)	Integrating mechanisms of pulmonary fibrosis	Journal of Experimental Medicine	Review	2011	1,069
3	Kany, Shinwan (15)	Cytokines in inflammatory disease	International Journal of Molecular Sciences	Review	2019	989
4	Butt, Yasmeen (16)	Acute lung injury: a clinical and molecular review	Archives of Pathology & Laboratory Medicine	Review	2016	695
5	Mueller, Amber L. (17)	Why does COVID-19 disproportionately affect older people?	Aging-US	Review	2020	618
6	Soy, Mehmet (18)	Cytokine storm in COVID-19: pathogenesis and overview of anti-inflammatory agents used in treatment	Clinical Rheumatology	Review	2020	576
7	Dolinay, Tamas (19)	Inflammasome-regulated cytokines are critical mediators of acute lung injury	American Journal of Respiratory and Critical Care Medicine	Article	2012	495
8	Gasse, Pamela (20)	IL-1R1/MyD88 signaling and the inflammasome are essential in pulmonary inflammation and fibrosis in mice	Journal of Clinical Investigation	Article	2007	417
9	Babelova, Andrea (21)	Biglycan, a danger signal that activates the NLRP3 inflammasome via Toll-like and P2X receptors	Journal of Biological Chemistry	Article	2009	396
10	Nieto-Torres, Jose L. (22)	Severe acute respiratory syndrome coronavirus envelope protein ion channel activity promotes virus fitness and pathogenesis	PLOS Pathogens	Article	2014	383

Ahmed et al. (13) conducted a comprehensive review of the Keap1/Nrf2/ARE signaling pathway, its downstream targets, and its effects in animal models of inflammatory diseases. The review also addressed the crosstalk between this pathway and the NF-κB pathway, as well as the regulation of the NLRP3 inflammasome by Nrf2. Additionally, the current landscape of anti-inflammatory phytochemicals and other agents that modulate the Nrf2/ARE signaling axis was summarized.

### An analysis of keywords

**FIGURE 5:**
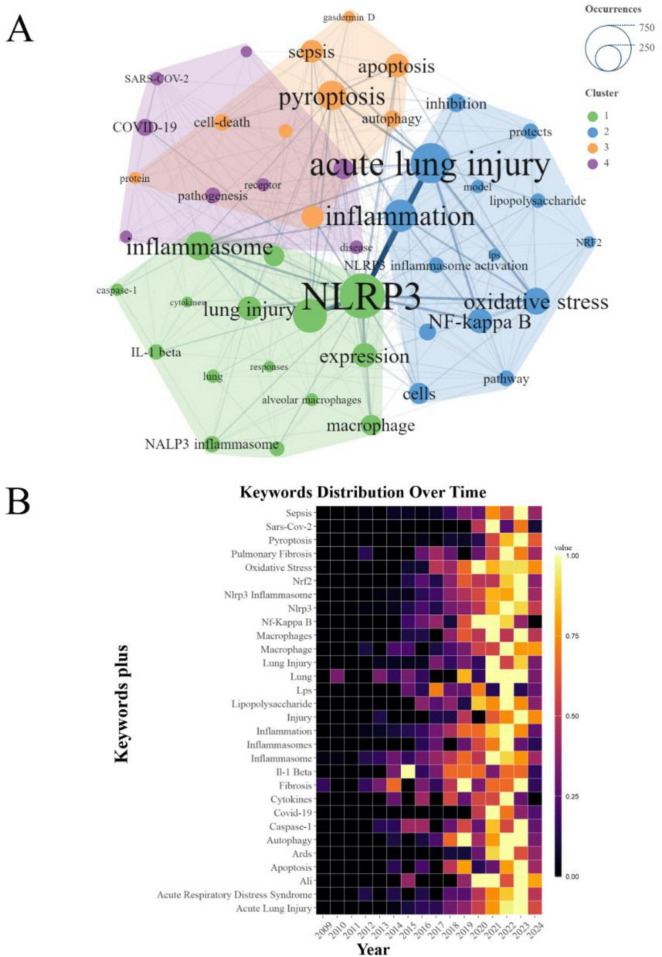
Analysis of keywords. **(A)** Clustering of the top 46 keywords with the highest number of occurrences. **(B)** Keywords heatmap of the top 30.

The comprehensive analysis of keyword co-occurrence among the 46 most prominent keywords in the PFALI research landscape has revealed four distinct thematic clusters. The cluster comprising keywords such as “acute lung injury,” “pyroptosis,” “NLRP3,” and “inflammation” demonstrated the most frequent and robust co-occurrence, underscoring the central importance of these conceptual focal points within the PFALI research domain (Figure 5A). Furthermore, a heatmap visualization of the top 30 keywords exhibiting the most dynamic emergence over time was presented in Figure 5B.

Building upon these synchronic and diachronic analyses of keyword patterns, the study also examined the broader trend topics that have gained prominence in PFALI research from 2000 to 2024. This longitudinal perspective illuminates the shifting research foci and emerging areas of scholarly interest within the field over the past two decades and into the near future. The research frontiers for the upcoming years have been identified as “iron,” “sirt1,” “repair,” and “alveolar macrophage pyroptosis” (Supplementary Figure 2), suggesting that these newly ascendant themes are poised to drive the next wave of advancements and discoveries within the dynamic and rapidly evolving PFALI research landscape.

## Discussion

Acute lung injury and its progression to acute respiratory distress syndrome (ARDS) represent the clinical manifestations of severe acute pulmonary inflammation. Globally, the mortality rate associated with ALI/ARDS can be as high as 35%–40% (16, 23). The common etiologies of ALI include infection, trauma, pulmonary contusion, and aspiration of gastric contents. The primary pathological features of ALI are a result of the severe inflammatory response in the lungs, leading to a series of lung tissue and cellular injuries, such as diffuse infiltration of mononuclear macrophages, destruction of the alveolar epithelial structure, increased microvascular permeability, and impaired gas exchange (24–26). However, the precise pathogenic mechanisms underlying the development of ALI remain unclear. Pyroptosis is a distinct form of cell death, primarily involving the classical inflammasome-induced pathway, the non-classical inflammasome-induced pathway, and the GSDM-related pathway. Pyroptosis is characterized by cell swelling and lysis, accompanied by the release of inflammatory cytokines such as interleukin-1β (IL-1β) and interleukin-18 (IL-18) (27–29). Growing evidence suggests that pyroptosis plays a crucial role in the pulmonary inflammatory response in ALI. Inhibiting pyroptosis is a promising therapeutic approach for ALI. To better elucidate the role of PFALI, this study conducted a comprehensive bibliometric analysis, examining a total of 1,495 publications, including 1,194 articles and 301 reviews, to assess the publication output on PFALI. Notably, China exhibited the highest publication output with 964 (64.48%) articles. Among the institutions analyzed, Central South University was found to be the most productive with 54 (3.61%) publications. The study findings indicate that Zhou, Yong had the highest publication records. International Immunopharmacology was the most productive journal with a total of 76 (5.09%) articles published. The research frontiers for the upcoming years have been identified as “iron,” “sirt1,” “repair,” and “alveolar macrophage pyroptosis.” Recent studies have focused on repairing cell functions through various mechanisms, such as the crosstalk between pyroptosis and ferroptosis (30), the SIRT1/NLRP3 pathway (31–33), alveolar macrophage (AM) pyroptosis (34–36). Cao et al. (30) found that crosstalk of pyroptosis, ferroptosis, and mitochondrial aldehyde dehydrogenase 2 mechanisms contributes to sepsis-induced lung injury in a mouse model. Zhang et al. (31) concluded that Apelin alleviates sepsis-induced ALI by modulating the SIRT1/NLRP3 pathway to inhibit endothelial cell (EC) pyroptosis, highlighting its clinical significance. Huang et al. (32) found that circVAPA overexpression targets miR-212-3p, negatively regulating SIRT1 and pyroptosis-related factors, which mitigates inflammatory damage in sepsis-induced ALI. Jin et al. (33) engineered VCAM-1-targeted nanostructured lipid carriers to deliver melatonin against acute lung injury via the SIRT1/NLRP3 pyroptosis signaling pathway. Zhao et al. (34) emphasized the protective role of ADAR1 in pulmonary macrophages against pyroptosis, suggesting targeting ADAR1/miR-21 signaling as a therapeutic option for sepsis-related lung injury. Liu et al. (35) demonstrated that MSC-derived exosomes effectively treat ALI by inhibiting AM pyroptosis and reducing inflammation through specific miRNAs and immunoregulatory proteins. Finally, Gong et al. (36) found that exosomal Tenascin-C, released from AECs under unresolved ER stress, exacerbates acute lung injury by intensifying sepsis-associated inflammatory responses, providing new insights into the cellular interactions involved in sepsis-induced ALI.

The research on PFALI has progressed and exhibited a global trend over time, and we have conducted a detailed bibliometric analysis of the literature. The key findings are as follows:

## The overall mechanism of pyroptosis in relation to ALI

### Pulmonary endothelial cell is involved in the pathogenesis of ALI

Pulmonary ECs line the inner wall of the lung microvasculature, forming the endothelial barrier (37). Pyroptosis of ECs and the subsequent release of IL-1β can induce injury and inflammation to the endothelial barrier, leading to the loss of endothelial integrity, increased vascular permeability, and the development of pulmonary edema in ALI (38, 39). Inflammatory caspases drive pyroptosis in acute lung injury, and the inhibition of EC pyroptosis may be an important therapeutic strategy for ALI. ECs injury and death occur at different stages of ALI, with lipopolysaccharide (LPS) inducing EC pyroptosis via the NLRP3/caspase-1/GSDMD pathway (40). NF-κB is a pro-inflammatory transcription factor that activates several genes, including NLRP3. Inhibiting the NF-κB-NLRP3-mediated pyroptosis in ECs can reduce LPS-induced ALI (41).

### Alveolar macrophage pyroptosis is involved in the pathogenesis of ALI

Alveolar macrophages are essential for innate immunity and host defense. The release of inflammatory cytokines by AMs and the subsequent regulation of other immune cells contribute to the persistent and excessive inflammatory response, which is a key factor in the pathogenesis of ALI. AMs pyroptosis is involved in the initiation of the inflammatory response in ALI (42, 43). The NLRP3-mediated pathway of AMs pyroptosis can trigger the release of HMGB1 from AMs. As a PAMP, HMGB1 further activates NLRP3 and caspase-1, creating a positive feedback loop that amplifies AM pyroptosis and worsens ALI severity (44). Pyroptosis can lead to organ injury resulting from ischemia/reperfusion injury. HMGB1 protein can activate the production of ROS and induce oxidative stress, triggering a series of inflammatory responses that contribute to the development of ALI. Recombinant HMGB1 (rH-MGBL1) can inhibit AMs pyroptosis and improve lung ischemia-reperfusion injury through the Keap1/Nrf2/HO-1 signaling pathway. This pathway suppresses the deleterious effects of pyroptosis and the associated inflammatory responses (45). Jiang et al. (46) identified the TRAF3-ULK1-NLRP3 axis as a critical regulatory pathway governing AMs pyroptosis in the context of ALI. The findings underscore the significance of this regulatory axis in ALI pathogenesis and suggest that targeting this pathway could represent an effective therapeutic strategy for ALI treatment. Liu et al. (35) reported that mesenchymal stem cell-derived exosomes (MSC-Exo) exhibited efficacy in the treatment of ALI by inhibiting AMs pyroptosis and attenuating the inflammatory response. The proposed mechanism involves pyroptosis-targeting miRNAs and immunoregulatory proteins delivered by MSC-Exo. These findings suggest that MSC-Exo may represent a promising new therapeutic option for the early management of ALI.

### Neutrophil pyroptosis is also involved in the pathogenesis of ALI

**FIGURE 6:**
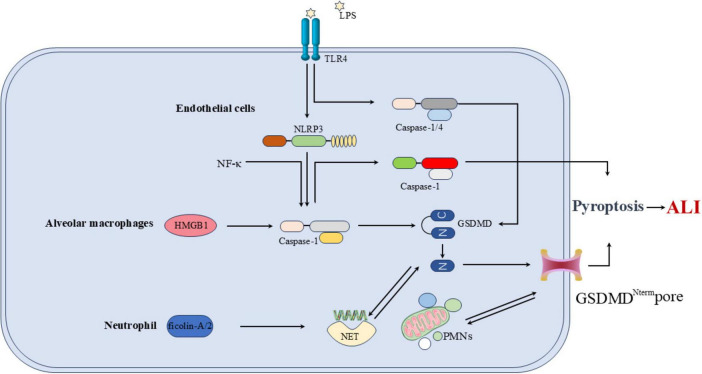
Overall mechanism of pyroptosis in relation to ALI. In ECs, ECs injury and death occur at different stages of ALI, with LPS inducing EC pyroptosis via the NLRP3/caspase-1/GSDMD pathway. NF-κB is a pro-inflammatory transcription factor that activates several genes, including NLRP3. In AMs, the NLRP3-mediated pathway of AMs pyroptosis can trigger the release of HMGB1 from AMs. As a PAMP, HMGB1 further activates NLRP3 and caspase-1, creating a positive feedback loop that amplifies AM pyroptosis and worsens ALI severity. In NE, ficolin-A/2 exacerbates severe lung injury by promoting NET formation, polymorphonuclear neutrophils (PMNs) are crucial in the development of sepsis-associated ALI.

In the LPS-induced ARDS mouse model and in ARDS patients, the cleavage of GSDMD is upregulated in neutrophils (NEs). The cleaved N-terminal fragment of GSDMD forms pores in the neutrophil membrane, promoting the release of neutrophil extracellular traps (NETs). NETs are web-like structures composed of chromatin fibers and granule-derived antimicrobial peptides and enzymes, which can capture and kill bacteria. The GSDMD fragments cleaved in neutrophils play a critical role in the development of ARDS through their involvement in NET formation (47). Huang et al. (48) found that ficolin-A/2 exacerbates severe lung injury by promoting NET formation, a process mediated by gasdermin D-induced pyroptosis. Polymorphonuclear neutrophils (PMNs) are crucial in the development of sepsis-associated ALI. PMN-derived exosomes contain miR-30d-5p, which targets cytokine signaling inhibitors SOCS-1 and SIRT1, activating the NF-κB pathway. This activation upregulates NLRP3 inflammasome expression in macrophages, leading to macrophage pyroptosis and promoting M1 polarization. The interactions between PMNs and macrophages contribute to the pathogenesis of sepsis-associated ALI (49). Pyroptosis of other cell types also contributes to the development of ALI. The long non-coding RNA NEAT1 mediates LPS-induced pyroptosis of BEAS-2B cells through the miR-26a-5p/Rho-associated coiled-coil containing protein kinase 1 (ROCK1) axis, contributing to the pathogenesis of sepsis-associated ALI (50) (Figure 6).

### Cellular pyroptosis is a therapeutic target for ALI

CircEXOC5 is a significantly upregulated circular RNA in ALI. It can suppress the Nrf2 transcription factor in LPS-stimulated mouse pulmonary microvascular ECs, thereby promoting cellular pyroptosis and attenuating LPS-associated ALI (51). Han et al. (52) revealed that irisin attenuates ALI by inhibiting the HSP90/NLRP3/caspase-1/GSDMD axis, reversing macrophage polarization, and reducing macrophage pyroptosis. These findings elucidate the mechanistic role of irisin in the treatment of ALI and ARDS. LL-37, also known as murine CRAMP, is a human antimicrobial peptide that plays a critical role in innate immune defense against sepsis through multiple mechanisms (53). Wang et al. (54) demonstrated that the antimicrobial peptide LL-37 protects against septic lung injury by modulating the expression of key pyroptotic mediators, including NLRP3, caspase-1, and GSDMD, as well as downstream inflammatory factors, in alveolar epithelial cells. 4-Hydroxynonenal (HNE) is a major endogenous product of lipid peroxidation. It can suppress the activation of the NLRP3 inflammasome in AMs, thereby reducing pyroptosis-related ALI in a sepsis mouse model (55). Shan et al. (56) showed that dynasore, a pharmacological agent, could alleviate LPS-induced ALI by regulating macrophage pyroptosis, suggesting a potential therapeutic strategy for ALI and ARDS. Natural products that inhibit cellular pyroptosis pathways can alleviate ALI. Emodin, a natural compound, can suppress NLRP3/caspase-1/GSDMD-mediated pyroptosis of AMs, thereby mitigating ALI (57). Citrulline provides protective effects against ALI by inhibiting ROS/NLRP3 inflammasome-dependent cellular pyroptosis (58). Zhang et al. (59) demonstrated that syringaresinol mitigates IgG immune complex-induced ALI by activating PPARγ and suppressing pyroptosis.

### Limitations

This bibliometric study offers a comprehensive assessment of the PFALI research landscape. The analysis evaluates the field’s overall progress, emerging trends, and influential references. The findings provide valuable guidance for researchers to prioritize the most impactful and recent literature. However, the study acknowledges certain limitations, including potential exclusion of recently published articles and the data source being constrained to the WoSCC. Additionally, inherent subjectivity in data interpretation is recognized. Nonetheless, this work serves as a representative snapshot of the current state and general trajectory of PFALI research, offering insights beneficial for researchers, clinicians, and policymakers in the domain.

## Conclusion

Cellular pyroptosis, a programmed cell death pathway associated with inflammation, plays a role in the inflammatory process of ALI. Our bibliometric analysis revealed that China has the highest publication output, with Central South University being the most prolific institution, and Zhou, Yong having the most publications. The journal International Immunopharmacology was the most productive. Key research frontiers for the coming years include “iron,” “sirt1,” “repair,” and “alveolar macrophage pyroptosis.” Targeting key factors in pyroptosis pathways, such as inflammatory caspases, offers new insights for reducing inflammation in ALI. Additionally, natural compounds that target this pathway show promising anti-inflammatory effects. However, the inflammatory response in ALI is influenced by various interconnected pathways, and the mechanisms of typical and atypical pyroptosis in lung tissue, along with targeted applications, require further study.

## Data Availability

The original contributions presented in this study are included in this article/Supplementary material, further inquiries can be directed to the corresponding author.

## Supplementary material

Supplementary Figure 1 Analysis of core journals. **(A)** Bibliometric coupling within journals. **(B)** Citations to each journal.

Supplementary Figure 2 Trend topics from 2000 to 2024.
