# Supplementary figures and images for: Bibliometric analysis of pyroptosis in pathogenesis and treatment of acute lung injury
Source: Front Med (Lausanne). 2025 Jan 22;11:1488796. doi: 10.3389/fmed.2024.1488796 (PMC11794077; doi:10.3389/fmed.2024.1488796)

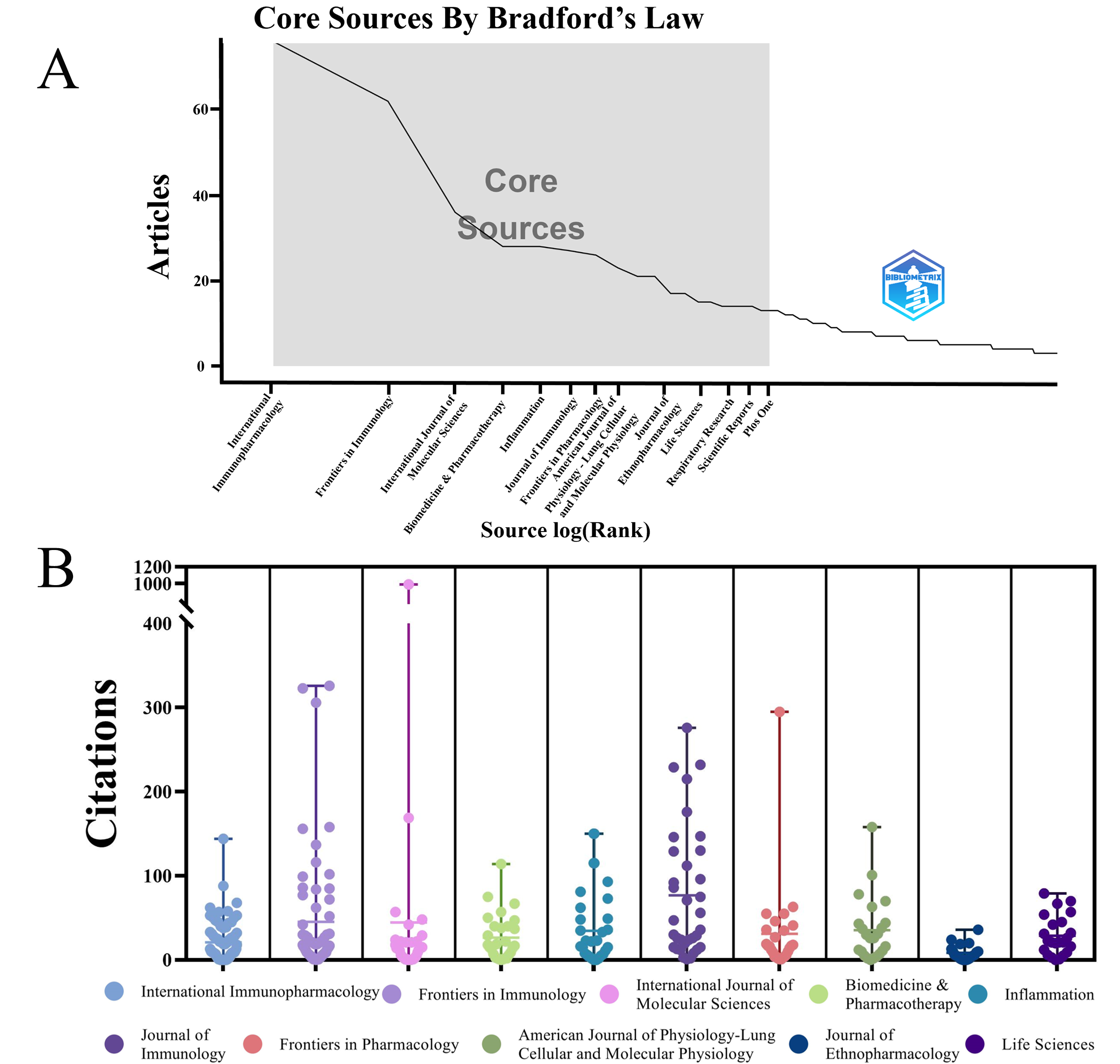

Supplement: Supplementary Figure 1 — Analysis of core journals. (A) Bibliometric coupling within journals. (B) Citations to each journal. [file Data_Sheet_1.zip › Supplementary figures/Supplementary figure 1.tif]

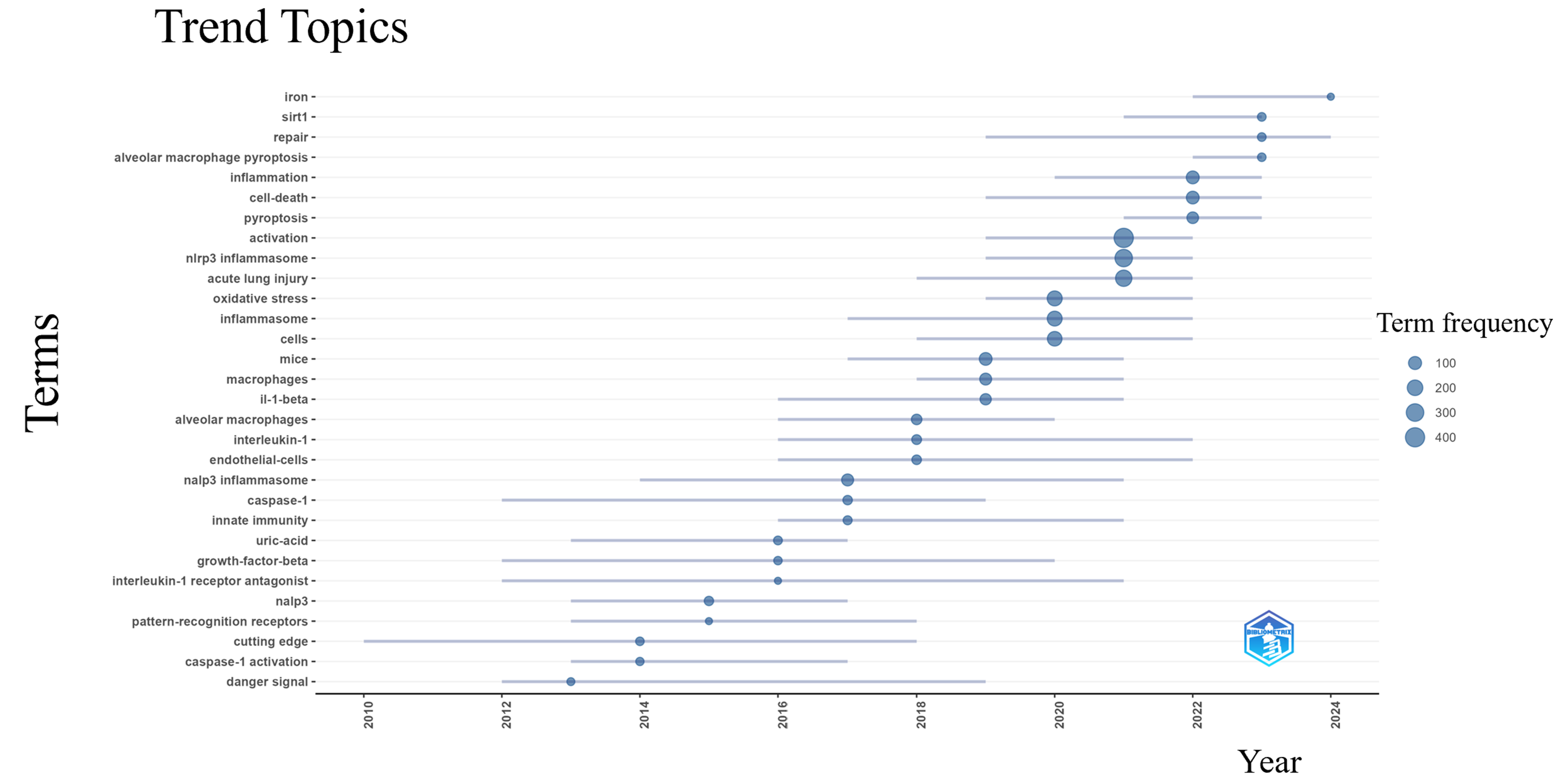

Supplement: Supplementary Figure 1 — Analysis of core journals. (A) Bibliometric coupling within journals. (B) Citations to each journal. [file Data_Sheet_1.zip › Supplementary figures/Supplementary figure 2.tif]
